# Expression of Concern: The mechanism of hsa-miR-424-5 combining PD-1 through mTORC signaling pathway to stimulate immune effect and participate in type 1 diabetes

**DOI:** 10.1042/BSR-20193800_EOC

**Published:** 2020-06-24

**Keywords:** hsa-miR-424-5p, signal molecule, mTORC signaling pathway, immune effect, type 1 diabetes, mechanism

The Editorial Office has been made aware of potential issues surrounding the scientific validity of this paper, hence has issued an expression of concern to notify readers whilst the Editorial Office investigates.

